# Multivessel Coronary Disease and Severe Atherosclerotic Aorta: Real-World Experience

**DOI:** 10.3390/medicina59111943

**Published:** 2023-11-02

**Authors:** Ivo Gasparovic, Panagiotis Artemiou, Andrej Domonkos, Branislav Bezak, Andrea Gazova, Jan Kyselovic, Michal Hulman

**Affiliations:** 1 National Institute of Cardiovascular Diseases, Clinic of Cardiac Surgery, Medical Faculty, Comenius University, 813 72 Bratislava, Slovakia; ivo.gasparovic@nusch.sk (I.G.); andrej.domonkos@nusch.sk (A.D.); branislav.bezak@nusch.sk (B.B.); michal.hulman@nusch.sk (M.H.); 2 Department of Pharmacology and Clinical Pharmacology, Medical Faculty, Comenius University, 813 72 Bratislava, Slovakia; aandreagazova@gmail.com; 3 5th Department of Internal Medicine, Medical Faculty Comenius, Comenius University Bratislava, 813 72 Bratislava, Slovakia; kyselovic@gmail.com; 4 Department of Pharmacology and Toxicology, University of Veterinary Medicine and Pharmacy in Kosice, 041 81 Kosice, Slovakia

**Keywords:** off-pump coronary artery revascularisation, hybrid revascularisation, atherosclerosis of the ascending aorta

## Abstract

*Background and Objectives*: Surgical revascularisation of patients with atherosclerosis of the ascending aorta remains a challenge. Different surgical strategies have been described in coronary surgical patients to offer alternative revascularisation strategies other than the conventional surgical revascularisation in patients unsuitable for it. The aim of this study is to compare the real-world outcomes between two groups of patients who underwent off-pump surgery (left internal mammary artery graft to the left anterior descending artery) or a hybrid with a percutaneous revascularisation procedure at a later stage. *Materials and Methods*: This is a single-centre retrospective observational study. Between the years 2010 and 2021, 91/6863 patients (1.33%) were diagnosed with severe atherosclerosis of the ascending aorta. All the patients were treated with off-pump revascularisation (91 patients), and the cardiologist would decide at a later stage whether the rest of the vessels would be treated with percutaneous revascularisation (25 patients). *Results*: There was no statistical difference in the various preoperative characteristics, except for coronary artery left main disease (30.30% vs. 64%; *p* = 0.0043). The two groups had no statistical differences in the perioperative characteristics and postoperative complications. The 1-, 5-, and 10-year mortality rates in the two groups were 6.1% vs. 0%, 59% vs. 80%, and 93.9% vs. 100%, respectively (off-pump vs. hybrid with percutaneous revascularisation procedure, *p* = 0.1958). *Conclusions*: Both strategies have high long-term comparable mortality. The off-pump surgery and the HCR procedure at a later stage may be solutions for these high-risk patients, but the target treatment should be complete HCR revascularisation during the index hospitalization.

## 1. Introduction

Surgical revascularisation of the patients with atherosclerosis of the ascending aorta remains a challenge. Conventional coronary artery bypass grafting (CABG) remains the gold standard of management for multivessel and left main coronary disease; however, manipulation of the aorta during the procedure in these patients is associated with an increased risk of postoperative complication, such as perioperative embolic stroke [1,2,3,4]. On the other hand, this group of patients is unsuitable for conventional surgery due to the severely atherosclerotic aorta (impossible to perform cannulation of the ascending aorta and applied cross-clamp), so other revascularisation strategies should be offered to them [1].

In order to avoid aortic manipulation and reduce morbidity (atheroembolism) and mortality, or to offer alternative revascularisation methods, the surgical strategy in high-risk or unsuitable patients for conventional CABG due to severely atherosclerotic ascending aorta is modified, and various techniques have been described and implemented. Such techniques include anaortic or no-touch off-pump surgery (OPCAB). Also, a body of evidence in the literature demonstrates the beneficial results of this strategy in high-risk patients [5].

Also, percutaneous coronary intervention (PCI) could be a reasonable alternative but not the first choice in patients with multivessel and left main coronary disease [3]. In patients with multivessel coronary disease, hybrid coronary revascularisation (HCR) is a safe and therapeutically effective alternative to CABG [6], as data also reveal a rather consistent trend toward a survival benefit of complete revascularisation (CR) over incomplete revascularisation (IR) in these patients [7]. Finally, failure to revascularise a non-left anterior descending (non-LAD) artery was not associated with a long-term adverse outcome [8].

In our institute, the preferred strategy in cooperation with the invasive cardiologists for patients with multivessel coronary disease and severe atherosclerosis of the ascending aorta for whom conventional CABG is indicated is OPCAB with a left internal mammary artery graft (LIMA) to the LAD coronary artery. The procedure is performed at a later stage if the patient is suitable for a PCI to the non-LAD targets after re-evaluation.

This study aims to compare the real-world outcomes between the two groups of patients who underwent only OPCAB with LIMA to LAD or HCR procedure with PCI at a later stage and to examine if the OPCAB procedure can be a solution for these high-risk patients.

## 2. Patients and Methods

**Figure 1:**
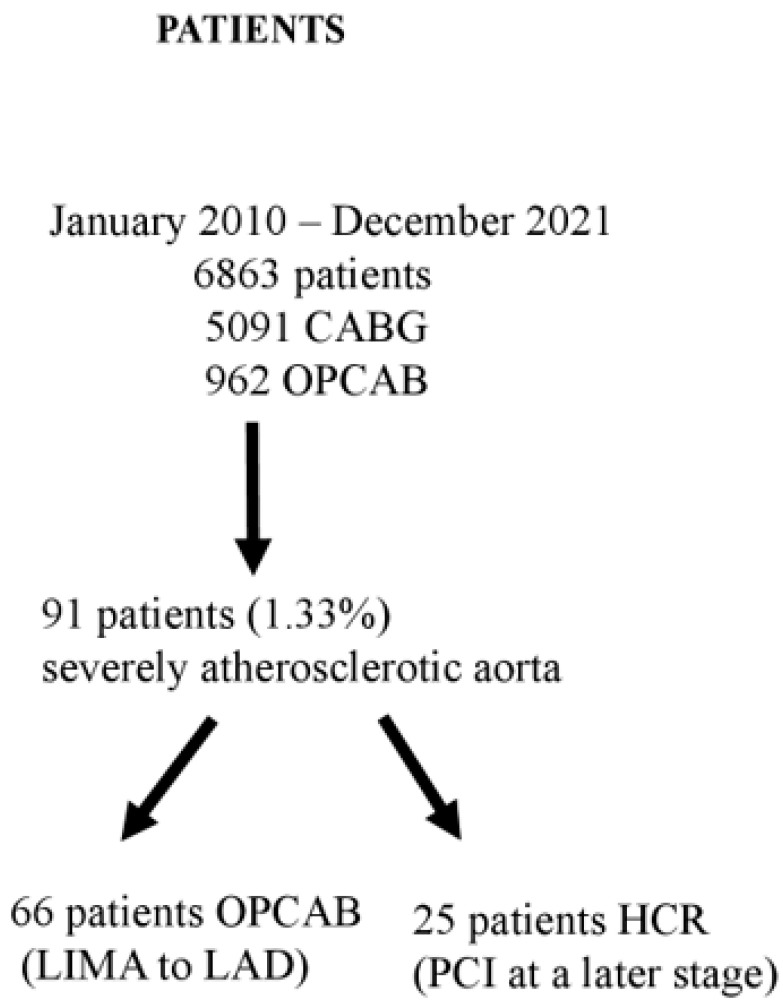
Flowchart showing the patient enrolment. CABG = coronary artery bypass graft; OPCAB = off-pump coronary artery bypass; LIMA = left internal mammary artery; LAD = left anterior descending artery; HCR = hybrid coronary revascularisation; PCI = percutaneous coronary intervention.

We designed a single-centre real-world retrospective observational study analysing patients from cardiosurgical registry (https://www.nczisk.sk) operated at the National Institute of Cardiovascular Diseases in Bratislava, Slovakia, in the period between 2010 and 2021. During that period, 6863 surgical revascularisation procedures (5901 CABG and 962 OPCAB) were performed. Ninety-one patients (1.33%) were diagnosed with severe atherosclerosis of the ascending aorta. This group of includes patients with multivessel coronary disease that were high-risk or unsuitable for conventional surgery. All coronary stenoses were ≥70%. The patients (91/962 (9.46%) underwent OPCAB procedures because it was impossible to perform cannulation of the ascending aorta and applied cross-clamp (Figure 1).

The diagnosis was made preoperatively using computed tomography (CT) or intraoperatively via inspection and palpation of the aorta. Epiaortic ultrasound is not routinely used in our practice. In order to achieve complete revascularisation, in cooperation with invasive cardiologists, HCR with PCI at a later stage is the preferred method of revascularisation. Firstly, a no-touch OPCAB with LIMA to LAD was performed, and the patients were, at a later stage, referred to the invasive cardiologist for re-evaluation and a PCI to the non-LAD targets. From the total number of 91 patients with severe atherosclerosis of the ascending aorta, 25 (26.3%) of them underwent HCR procedure with PCI at a later stage (group 2). The rest of the patients underwent only OPCAB with LIMA to LAD (group 1). The decision to perform an HCR procedure with PCI at a later stage was made by the invasive cardiologists who re-evaluated the patient and was based on the clinical status of the patient (angina pectoris or myocardial infarction), the area of the jeopardised myocardium, the characteristics of the coronary vessel and stenosis, and the complexity of the procedure.

In our institute, every patient over 70 years old undergoes a preoperative non-contrast CT of the aorta to rule out severe calcifications.

Baseline clinical preoperative, perioperative, and postoperative data were obtained from the cardiosurgical registry (https://www.nczisk.sk). The two groups of patients were compared using clinical scores like Euroscore II, Canadian Cardiovascular Society (CCS) grade, New York Heart Association (NYHA) class, and the SYNTAX (Synergy Between Percutanneous Coronary Intervention with Taxus and Cardiac Surgery) score. Additional follow-up postoperative data were obtained from patient documentation. Mortality data were obtained upon request from The Health Care Surveillance Authority registry (https://www.udzs-sk.sk/en).

The OPCAB and PCI procedures were performed in a standard way. The mean follow-up time was 1282.19 ± 962.93 days. Patient informed consent was obtained.

## 3. Statistical Analysis

Statistical analysis was performed using GraphPad Prism version 5.00 (GraphPad Software, San Diego, CA, USA). All variables were expressed as mean ± standard deviation, qualitative variables as numbers and percentages. The Shapiro–Wilk method was used for normality testing. Analysis of variance was performed, followed by unpaired *t*-test or χ^2^ test (Fisher’s exact test), Tukey’s multiple comparison test, and Mann–Whitney test when the variables did not show normal distribution; survival data were analysed with the log-rank (Mantel–Cox) test. *p*-value (*p) of <0.05 was considered as statistically significant.

## 4. Results

The preoperative characteristics of the two groups are shown in Table 1. Mean age in the two groups was 72.15 ± 6.04 and 71.80 ± 8.73 years, respectively. The majority of the patients in both groups were men, 85% in group 1 and 92% in group 2. The incidence of left main disease was significant prevalent in group 2 (group 1 30.30% vs. group 2 64% *p* = 0.0043), but the two groups had comparable SYNTAX score. Also, patients in both groups had comparable risk of mortality (Euroscore II mean ± SD, group 1 3.19 ± 2.69 vs. group 2 2.82 ± 1.47; *p* = 0.7671).

Comparing the perioperative characteristics of the patients, there were no significant statistical differences between the two groups. The perioperative characteristics are shown in Table 2.

There was no statistical difference between the various postoperative complications between the two groups. Moreover, in group 2, no post-procedural complications were observed after the PCI procedures. The mean time of hospitalisation between the first and second groups was 21 ± 46 vs. 13 ± 4 (*p* = 0.4395) days, respectively (Table 3).

The median time from OPCAB to PCI in group 2 was 32 (0–810) days. The vessel with the most revascularisation procedures was the circumflex artery (LCX) with 16 procedures, followed by the right coronary artery (RCA) with 8 revascularisation procedures. Forty-two coronary artery vessels were not revascularised (LCX = 22, RCA = 20). Data from group 2 are shown in Table 4.

**Figure 2:**
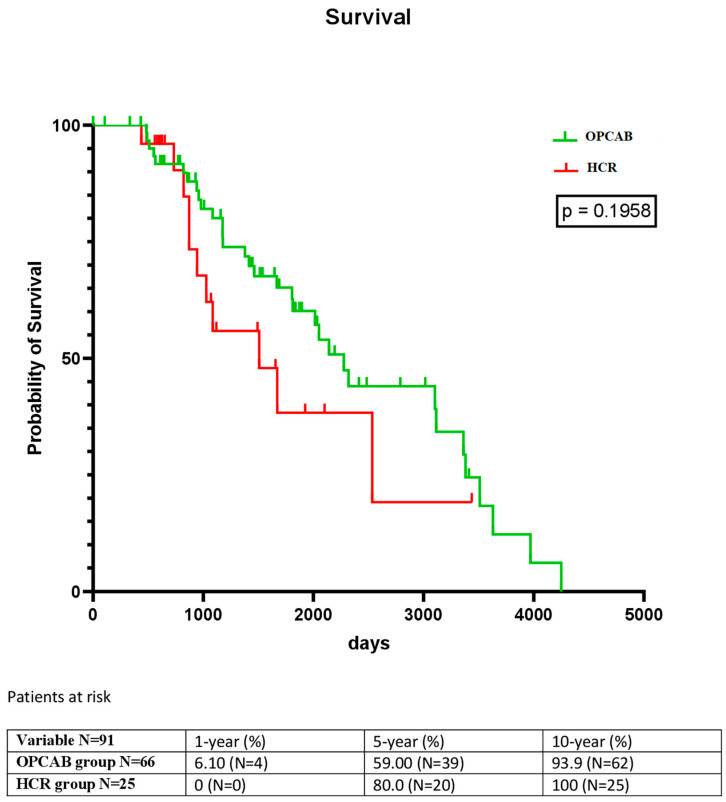
Kaplan–Meier analysis showing the long-term survival in both groups.

The hospital mortality was 4.55% in group 1 vs. 0% in group 2 (*p* = 0.7634). The 1-, 5-, and 10-year mortality rates in the two groups were 6.1% vs. 0%, 59% vs. 80%, and 93.9% vs. 100%, respectively. The mean time from OPCAB or PCI to death was 1267.09 ± 850.64 days. The long-term survival is shown in Figure 2 (*p* = 0.1958).

## 5. Discussion

Surgical revascularisation of high-risk or unsuitable patients for conventional CABG patients with atherosclerosis of the ascending aorta remains a challenge. Different surgical strategies for treating coronary surgical patients aiming to prevent embolic stroke, as well as alternative revascularisation strategies other than the conventional CABG unsuitable for conventional surgical revascularisation patients were described. In a review study, Knoll et al. [3] recommend level 2 evidence, off-pump surgery as the preferred method in patients with severe aortic atherosclerosis in cases where experts are available and complete revascularisation can be achieved. Other strategies recommending level 3 evidence to reduce stroke rate are as follows: minimizing clamping (single clamp on-pump and off-pump with clamp-less facilitating or no touch) and axillary artery cannulation as an alternative or replacement of the severely atherosclerotic aorta if necessary [3].

Another technique for complete surgical myocardial revascularisation without any handling of the ascending aorta is the use of Y or T composite grafts, in which venous or arterial grafts are anastomosed to the LIMA. The use of composite grafts is described in the literature as a safe approach, with evidence that the LIMA is capable of adapting to provide blood flow to more than 1 coronary artery [9,10].

For patients with severe ascending aortic calcification, avoiding the use of side clamps is one of the most important means to prevent neurologic complications after CABG. To solve this problem, many commercial auxiliary devices like Enclose (Novare Surgical Systems, Cupertino, CA) or Heartstring (Maquet Cardiovascular, Rastatt, Germany) have been introduced in recent years to provide a clamp-less proximal anastomosis. Wang C et al. described an alternative technique to create a clamp-less proximal anastomosis using a Foley catheter and polypropylene suture. However, even the minimal aortic manipulation resulting from this technique still represents a risk of atheroembolism [11].

Percutaneous coronary interventions could be an alternative in patients with an indication for CABG, but not as a first choice. Data analysis from several trials of randomized patients to PCI or CABG showed a lower perioperative stroke risk after PCI [12]. However, the 30-day stroke rate of no-touch OPCAB was lower when compared to PCI in a network meta-analysis [13]. Moreover, PCI is associated with lower survival in patients with an indication for CABG [14].

There is a body of evidence in the literature that indicates the role of OPCAB in select high-risk patients. In a randomised trial in patients with EuroSCORE > 6, Lemna et al. [15] showed that operative mortality was significantly lower in the OPCAB group. Moreover, few meta-analyses in elderly patients have confirmed that OPCAB offers benefits as compared with conventional CABG [16,17].

By taking all the above factors into consideration, in our institute, in patients with severe aortic atherosclerosis who are indicated for CABG, in order to avoid aortic manipulation (aortic cross-clamping, partial cross-clamping), we recommend no-touch aorta OPCAB with LIMA to LAD, and after evaluation for PCI suitability, PCI should be performed in a later stage with drug-eluting stents (DES) to the non-LAD targets. Hybrid coronary revascularisation avoids aortic manipulation and offers the durability and survival advantages of the LIMA to the LAD graft and DES to non-LAD targets [6].

Theoretically, HCR combines the risk of stroke in major surgery with the risk of plaque disruption through intra-aortic manipulation [18]. However, our results showed that selected patients with severe aortic atherosclerosis may be good candidates for the HCR procedure, while the OPCAB procedure did not increase the risk of perioperative stroke. Moreover, no perioperative complications or stroke were observed after the PCI procedures. In our study, there was no statistical difference in the stroke rate between the OPCAB and HCR group. In the group analysis, only 1 (1.4%) patient from the OPCAB group had a perioperative stroke.

In the literature, the results are contradictory. A network meta-analysis showed a lower rate of stroke in simultaneous HCR compared to staged HCR or conventional CABG [19]. This contrasts with the results of the randomized controlled trials, which did not observe a lower stroke rate [19].

In the same context, Nazer RI et al. [20] proposed the brain-before-heart strategy for coronary artery grafting in severely atherosclerotic aorta. The authors stated that early detection and individualized therapeutic strategies are effective in reducing atheroembolic brain injury and are associated with better prognosis.

It was observed that from the 95 patients with severely atherosclerotic aorta who were referred for HCR procedure, only 25 patients underwent PCI to the non-LAD targets, with the rest of them, 70 patients, having incomplete revascularisation with only OPCAB, LIMA to LAD. The decision to perform the HCR procedure was made by the invasive cardiologists and was based on the clinical status of the patient, the area of the jeopardised myocardium, the characteristics of the coronary artery and stenosis, and the complexity of the procedure.

In our study, despite the patients’ qualification for hybrid revascularisation, complete revascularisation was achieved relatively rarely. Complicated multivessel disease remains a challenge for complete revascularisation, but its benefits have been reported in the literature. In the SYNTAX trial [21], in both the PCI and the CABG groups, cardiac death and all-cause mortality were significantly lower among patients who received complete revascularisation. Similar results were found from the British Columbia cardiac register [22], where in stable patients with multivessel disease, complete revascularisation was an independent predictor of long-term survival, and this benefit was specifically seen in higher risk patients.

The incomplete revascularisation in the OPCAB and the HCR groups had an impact on the patients’ survival rate. In accordance with the evidence in the literature where complete revascularisation had a survival benefit over incomplete revascularisation [7], our results showed that both groups of patients had a statistically similar 5-year decreased survival rate. In our study, the survival benefit and the influence of the LIMA graft on survival after CABG was not confirmed as it was in the literature [23]. Moreover, contrary to our results, Fefer P et al. showed that failure to revascularise a non-LAD artery was not associated with a long-term adverse outcome [8].

Increasing age is a risk factor associated with the development of postoperative stroke [4], and non-contrast preoperative chest computed tomography (CT) or CT angiography are useful tools for screening aortic calcifications in these high-risk patients. In our institute, there is a consensus that every patient over 70 years old should have a preoperative non-contrast CT of the aorta to rule out severe calcifications. Contrary to our policy, some institutes recommend performing preoperative screening chest CT in patients >60 years old [24].

In patients who are indicated for CABG, based on the CT scan finding (severe atherosclerosis of the ascending aorta), the surgical strategy is changed, and the patients are referred for HCR procedure (firstly OPCAB to LAD and later PCI to the non-LAD targets if are eligible).

The decrease in postoperative stroke and mortality by the preoperative CT is well described in the literature [25,26]. On the other hand, in a randomized trial, Knoll et al. [27] reported that preoperative non-contrast CT in cardiac surgery candidates did not influence the surgical approach nor the incidence of perioperative stroke compared with standard of care.

The long-term mortality in our study is higher than the mortality which is reported in other different coronary artery revascularisation studies [28]. Our group included only high-risk patients with severely atherosclerotic ascending aorta and generalized atherosclerosis, which is a risk factor for earlier mortality [29].

Finally, although we acknowledge the benefits of multivessel OPCAB procedure, we believe that for this group of patients with severely atherosclerotic aorta, the HCR procedure is a safer solution, taking into account the outcomes of the OPCAB procedure that were described in different studies (decreased graft patency and survival) [30]. Moreover, in our institute, the preferred method of revascularisation is on-pump CABG.

Inflammation plays a key role in the development of atherosclerosis and coronary artery disease [31]. Inflammation and immune cells CD4+ not only take part in cardiac injury [32,33], but also have an essential role in promoting cardiac homeostasis and injury repair, but these repair processes also increase “bystander damage” that overreact to injury [34]. In our study, we did not check any inflammation markers, but all the patients of the study received statin treatment for hyperlipidaemia. The statins, with their anti-inflammatory effect, may help prevent cardiovascular events in the future. Furthermore, anti-inflammatory interventions specifically blocking the cytokine pathways would reduce the risk of MI and stroke [31].

In our cohort, LM disease was more prevalent in the HCR group. This difference may have impacted the long-term results of the treatment, but on the other hand, the severity of the coronary artery disease expressed as SYNTAX score was comparable between the two groups. Both groups had highly severe coronary artery disease, expressed as high SYNTAX score, which is an independent predictor of long-term mortality [35], and this may be the reason why, in our study, both groups had a high long-term mortality.

The limitations of the study are the following: First is the retrospective design of the study and the low number of patients. Moreover, patients in the hybrid group who underwent PCI at a later stage were not randomly selected, and the decision for revascularisation was left to the cardiologists. Even in the hybrid group, half of the vessels were left untreated, and it is finally unclear in how many cases the revascularisation was complete. Moreover, we could not compare the OPCAB procedure with similar patients whose ischemic heart disease was treated with techniques other than OPCAB (optimal medical therapy, multivessel PCI) because we did not have such data to compare. A propensity match analysis was not performed because the two groups were similar in terms of preoperative characteristics. In order to have definite answers concerning the management of these high-risk patients a prospective randomized study should be designed.

## 6. Conclusions

Based on our results, both strategies have high long-term comparable mortality. Although the high mortality, the off-pump surgery and the HCR procedure in selected cases may be solutions for these high-risk patients. The goal treatment should be complete revascularisation, and an early HCR procedure with complete revascularisation during the index hospitalization would decrease the mortality of these patients.

## Figures and Tables

**Table 1:** Preoperative characteristics of the patients in the OPCAB and HCR groups.

Variable *n* = 91	OPCAB Group *n* = 66 (%)	HCR Group *n* = 25 (%)	*p* Value
Age—(years) (mean ± SD)	72.15 ± 6.04	71.80 ± 8.73	0.828
Gender	M = 56 (85%); F = 10	M = 23 (92%); F = 2	0.5 ^c^
BMI—(kg/m^2^) (mean ± SD)	27.75 ± 4.12	28.80 ± 3.61	0.2667
Euroscore II (mean ± SD)	3.19 ± 2.69	2.82 ± 1.47	0.7671
Ejection Fraction (mean ± SD)	49.38 ± 10.72	49.32 ± 10.44	0.9813
CCS grade (mean ± SD)	2.34 ± 1.06	2.36 ± 1.17	0.8728
NYHA class (mean ± SD)	2.06 ± 0.91	1.96 ± 1.04	0.6461
Coronary artery disease			
LM disease	20 (30.30%)	16 (64%)	0.0043 ^b^
2-vessel disease	18	5	0.5937 ^c^
3-vessel disease	42	19	0.3234 ^c^
SYNTAX score (mean ± SD)	20.47 ± 7.33	21.88 ± 5.91	0.3473 ^b^
Diabetes mellitus	34	10	0.3566 ^c^
Oral antidiabetic treatment	22	5	0.3048 ^c^
Insulin treatment	9	2	0.7209 ^c^
Smoking	19	11	0.2132 ^c^
Hypertension	66	25	N/A
Hypercholesterolemia/statin users	53	18	0.4064 ^c^
Hepatic disease (*n*)	8	0	0.1010 ^c^
Renal disease (*n*)	6	3	0.7019 ^c^
Chronic obstruction pulmonary disease (*n*)	7	3	>0.9999 ^c^
Peripheral vascular disease (*n*)	13	4	0.7724 ^c^
Preoperative stroke (*n*)	10	4	>0.9999 ^c^
Atrial Fibrillation (*n*)	7	1	0.4374 ^c^

OPCAB = off-pump coronary artery bypass; HCR = hybrid coronary revascularisation; BMI = body mass index; CCS = Canadian Cardiovascular Society; NYHA = New York Heart Association; SYNTAX = Synergy Between Percutaneous Coronary Intervention with Taxus and Cardiac Surgery; LM = left main; N/A = not available; M = male; F = female. Statistical analysis: ^b^ = Fisher´s exact test; ^c^ = Chí test; other values—unpaired *t*-test; SD = standard deviation.

**Table 2:** Perioperative characteristics of the patients in the OPCAB and HCR groups.

Variable *n* = 91	OPCAB Group *n* = 66 (%)	HCR Group *n* = 25 (%)	*p* Value
Conduit used			
LIMA	65	25	>0.9999 ^c^
SVG	1	0	>0.9999 ^c^
Non-revascularized vessels			
Right coronary artery	52	22	0.3817 ^c^
Circumflex artery	49	20	0.7845 ^c^
Blood loss (ml) (mean ± SD)	651.5 ± 235.7	690 ± 332.9	0.5385
Transfusion (PRC units)	6 (10%)	5 (25%)	0.1666 ^c^
Inotrope use	29	10	0.8148 ^c^
Intubation time (hours) (median/IQR)	10/11	18/14	0.9706 ^a^
Reintubation	3	0	0.5586 ^c^
ICU stay (hours) (mean ± SD) (median/IQR)	72/27	72/49.5	0.9348 ^a^

OPCAB = off-pump coronary artery bypass; HCR = hybrid coronary revascularisation; LIMA = left internal mammary artery; SVG = saphenous vein graft; PRC = packed red cells; SD = standard deviation; ICU = intensive care unit; ^c^ = Chí test; other value—unpaired *t*-test; ^a^ = Mann–Whitney test.

**Table 3:** Postoperative complications of patients in the OPCAB and HCR groups.

Variable *n* = 91	OPCAB Group *n* = 66 (%)	HCR Group *n* = 25 (%)	*p* Value
Stroke	1	0	>0.9999
Pneumonia	6	2	>0.9999
Urinary track infection	3	2	0.6126
Renal failure	4	0	0.5720
Postoperative atrial fibrillation	1	1	0.4762
Bleeding/re-exploration	0	0	N/A
Post-operative MI	2	1	>0.9999
Sternal wound infection	2	0	>0.9999
Hospital stay (days) (mean ± SD)	14/7	14/6	0.6685 ^a^
Hospital mortality	3 (4.55%)	0 (0%)	0.7634

OPCAB = off-pump coronary artery bypass; HCR = hybrid coronary revascularisation; MI = myocardial infarction; N/A = not available; SD = standard deviation. Other values—Chí test; ^a^ = unpaired *t*-test.

**Table 4:** PCI data of the hybrid procedures.

Variable	HCR Group *n* = 25
Non-revascularized vessels	42
Right coronary artery	20
Circumflex artery	22
PCI vessels	24
Right coronary artery	8
Circumflex artery	16
Time from OPCAB to PCI (days) (mean ± SD), median	123.6 ± 209.4, 32 (0–810)

OPCAB = off-pump coronary artery bypass; HCR = hybrid coronary revascularisation; PCI = percutaneous coronary intervention; SD = standard deviation.

## Data Availability

The data presented in this study are available on request from the corresponding author.

## Abbreviations

OPCAB = off-pump coronary artery bypass; CABG = coronary artery bypass grafting; LIMA = left internal mammary artery; SVG = saphenous vein graft; PRC = packed red cells; SD = standard deviation; ICU = intensive care unit; HCR = hybrid coronary revascularisation; BMI = body mass index; CCS = Canadian Cardiovascular Society; NYHA = New York Heart Association; SYNTAX = Synergy Between Percutaneous Coronary Intervention with Taxus and Cardiac Surgery; LM = left main; N/A = not available; M = male; F = female; MI = myocardial infarction; LAD = left anterior descending artery; LCX = left circumflex artery; RCA = right coronary artery; PCI = percutaneous coronary intervention; DES = drug eluting stent.
